# Choosing how to decide: empowering patients for shared decision-making in end-of-life care

**DOI:** 10.1007/s11019-026-10389-5

**Published:** 2026-08-18

**Authors:** Nicholas Abernethy

**Affiliations:** Sage School of Philosophy, Cornell University, Ithaca, NY USA

**Keywords:** Shared decision-making, End-of-life care, Undue influence, Autonomy, Clinical ethics

## Abstract

Shared Decision-Making (SDM) is a process wherein patients and clinicians make healthcare decisions collaboratively. There are two main versions of SDM. The first is narrow SDM, wherein the clinician’s role is limited to things like providing information and eliciting (without challenging) the patient’s preferences. The second is broad SDM, wherein the clinician participates in the decision-making in various additional ways (e.g., by providing recommendations). In this paper, I argue that there are cases where a patient would not be wronged by a clinician letting them choose from a range of versions of SDM – including narrow SDM and types of broad SDM – for an end-of-life treatment decision. This paper’s structure is as follows. First, I explain my proposal. Second, I provide a brief rationale for it. Third, I thoroughly defend it from the likeliest objection, which holds that clinicians in the relevant cases should offer only narrow SDM because broad SDM would unduly influence patients. This paper’s originality comes from (i) presenting a new and uniquely detailed proposal for when and how to offer versions of SDM and (ii) exploring the ethical implications of a patient’s choice of a version, understood as an act of consent. The main purpose of my proposal’s many details is to maximize how much the SDM would advance patient autonomy (and, secondarily, patient wellbeing).

## Introduction

Imagine a physician telling a terminally ill patient that they believe that the patient’s values favor choosing assisted dying. Could this be done in a way that does not wrong the patient? With many provisos, this paper will say yes. This is because this paper will argue that there are cases where a patient would not be wronged by a clinician (e.g., a physician or a nurse practitioner) letting them choose from a range of versions of Shared Decision-Making (SDM) – including a version with recommendations – for an End-Of-Life (EOL) treatment decision.^1^ There are many definitions of SDM in the academic literature, but this paper uses the following: “Shared decision making is a process during which clinicians and patients collaborate to make health decisions, considering both the best available evidence and patients’ preferences” (Politi et al. 2013, p. 18).^2^

For this paper’s purposes, there are two main versions of SDM (both of which are offered on this paper’s proposal). The first is narrow SDM. As Vikki Entwistle et al. explain,

Narrow understandings of shared decision-making are characterized by an emphasis on engaging patients in choice by informing them about healthcare options then eliciting and respecting (not interfering with) their preferences… Narrow understandings of shared decision-making promote a division of labour in which… patients work out which option they prefer… Any challenging of patients’ preferences is restricted to checking patient’s factual understanding and reiterating or providing more information (Entwistle et al. 2012, 416–417).

In narrow SDM, the clinician does not do things like provide recommendations (Entwistle et al. 2012).

The second main version is broad SDM, which involves the clinician participating in the decision-making in various ways in addition to providing information and eliciting preferences. As James and Marcia Childress explain,

broad models of SDM… acknowledge that health professionals might need to support patients’ discernment, criticism, and deployment of their own values and preferences – for instance, through recommendations and probing questions. If the decision-making process includes a more collaborative conversation, practitioners can invite patients to explore their preferences, expectations, and rationales. The 2 parties can then partner in determining the patient’s best choices in the circumstances (Childress and Childress 2020, p. 425).

Other communicative techniques that exemplify broad SDM include helping the patient conceptualize weighing the pros against the cons, as well as suggesting other ways to view their situation (Elwyn et al. 1999; Epstein and Gramling 2013). In short, “Division of labour is less strict. Dialogue is more open ended” (Entwistle et al. 2012, p. 417). Admittedly, my description of broad SDM is relatively fuzzy, and this is because broad SDM can take many forms (as I will explain).

This paper’s structure is as follows. First, I explain my proposal for when and how to offer “SDM options” (by which I mean options for exactly how SDM happens). Second, I provide a brief rationale for my proposal. Third, I thoroughly defend it from the likeliest objection, which holds that clinicians in the relevant cases should offer only narrow SDM because broad SDM would unduly influence patients. This objection reflects the fact that broad SDM is more controversial than narrow SDM (Cribb and Entwistle 2011; Childress and Childress 2020).

This paper focuses on EOL care for two main reasons (both of which will become clearer as the paper proceeds). First, EOL care very often involves the type of treatment decision that is widely recognized as best suited for broad SDM, namely, decisions that are high-stakes, highly value-sensitive, and lacking a clinically superior treatment option (i.e., an option that is better than the others according to the empirical evidence) (Rabben et al. 2024). Second, despite the first reason, broad SDM is especially controversial for EOL care (Zhou and Shelton 2020), partly because concerns about undue influence are especially salient for EOL care (Lim 2003). As Micah Prochaska and Daniel Sulmasy summarize, “Recent literature has challenged the idea that physician recommendations should be part of shared decision making at the end of life” (Prochaska and Sulmasy 2015, p. 693). At least at first glance, this extra controversy makes sense. For example, recommending assisted dying seems more problematic than recommending an appendectomy. This is reflected in Canadian law, which prohibits healthcare professionals from persuading patients to request assisted dying (CAMAP 2022).^3^ Ultimately, if this paper succeeds in defending my proposal for many of the treatment decisions for which it would be the most controversial, then it is probably defensible for many treatment decisions for which it would be less controversial.

Other theorists have already argued that patients should be allowed to choose from a range of SDM options for treatment decisions generally (Dowie 2002; Kon 2010),^4^ and other theorists have discussed SDM in EOL care (Foo et al. 2024; Pujol et al. 2025). So, this paper’s originality comes from (i) presenting a new and uniquely detailed proposal for when and how to offer SDM options and (ii) exploring the ethical implications of a patient’s “SDM choice” (by which I mean their selection of an SDM option), understood as an act of consent. The main purpose of my proposal’s many details is to maximize how much the SDM would advance patient autonomy (especially by enhancing rationality and avoiding undue influence) and, secondarily, increase patient wellbeing.

Lastly, it is worth clarifying that this paper is about the ethics of clinician actions rather than the ethics of institutional policies governing clinician actions. I take no position on whether institutions like hospitals should formally permit the actions that I defend on ethical grounds. After all, there are other considerations (e.g., enforceability concerns) that must be factored into institutional policies regarding SDM. However, I maintain that my analysis is highly relevant to the policy questions.

## My proposal

### The cases in question

I begin by identifying the cases in which I am defending a clinician offering a range of SDM options for an EOL treatment decision. These cases have seven features.^5^ First, the EOL treatment decision is high-stakes, highly value-sensitive, and lacking a clinically superior treatment option. For example, imagine a patient whose options are palliative care, assisted dying, and a life-prolonging intervention with severe adverse effects. Second, the clinician has a knowledgeable and trusting relationship with the patient, which facilitates SDM (Abdul-Razzak et al. 2014; Joseph-Williams et al. 2014). Often, this will be a long-term relationship. Third, the clinician has the skills (e.g., regarding communication and critical analysis) that are necessary for doing SDM well.^6^ Fourth, the clinician has enough time to do SDM well. Fifth, the patient is emotionally prepared for the SDM choice. This can require waiting until they have recovered from the shock of a diagnosis (Joseph-Williams et al. 2014). Sixth, the patient has decision-making capacity for both the SDM choice and the treatment decision.^7^ Although there are disagreements over precisely defining decision-making capacity, theorists generally agree that it involves the abilities to communicate a decision, understand the relevant facts, appreciate the nature of the decision, and rationally reason regarding the decision (Hawkins and Charland 2020).^8^ Seventh, the clinician has already provided the patient with basic clinical information about their medical condition and their treatment options, and the clinician tried their best to do so neutrally and non-directively.

To be clear, the presence of this seventh feature means that, on my proposal, some narrow SDM occurs *before* the patient chooses from the SDM options. My rationale for this is that the information in question is necessary for the patient to make an informed choice from the SDM options. It is beyond the scope of this paper to explore the debate over whether patients have a right to refuse decision-relevant information (and thus to forgo the preliminary narrow SDM); this paper applies only in cases where the preliminary narrow SDM has already happened. Also, I leave open whether my proposal is best suited for specialists (e.g., oncologists) or generalists (e.g., family doctors).

Some readers may object that there are few cases in which all seven features apply. I have two responses. First, many of the features could be – and should be – made more commonly applicable. In particular, clinicians should receive more and better communication training, especially for EOL contexts (Granek et al. 2013; Visser et al. 2014), which could have the added benefit of making many of the other features apply in more cases. For example, better communication can improve patient-clinician relationships (Borhani-Haghighi 2022). If those in power heed the existing calls regarding communication training,^9^ there will be an increase in the number of cases that fall within this paper’s purview.

Second, I am defending my proposal in cases where all seven features apply, but this does not mean that I am saying that my proposal is indefensible in cases where some of the features do not apply. Within EOL contexts, I am focusing on cases that are closer to being ideal for my proposal, and I am leaving open how non-ideal cases can get before they cease to be suitable for my proposal. For example, my proposal may be defensible in some cases where a patient is emotionally unprepared for the SDM choice but not in a way that precludes decision-making capacity.

### The SDM options

There are many possible approaches to offering a patient a range of SDM options. For example, a clinician could ask them to choose a point “on the shared decision-making continuum” (Kon 2010, p. 904) or a cell in a “multidimensional [SDM] matrix” (Dowie 2002, p. 16). Approaches can go wrong by giving a patient either too many options (and thereby overwhelming them) or too few options (and thereby insufficiently aligning the SDM with their preferences). So, definitively determining the best approach would require empirical research (e.g., on the point of diminishing returns). Unfortunately, this research does not exist, so I will propose an approach that seems to strike a good balance, unlike a continuum with only one dimension (Kon 2010) and a complex matrix with 162 permutations (Dowie 2002). For the sake of brevity, I will present my approach in general terms (rather than in terms of the previously specified EOL treatment decisions), but this should not be misinterpreted as an endorsement of using my approach for all treatment decisions. For example, it would be a colossal waste of time to use my approach before recommending emergency surgery for a ruptured appendix.

On my approach, the initial question that the clinician asks the patient is whether they would prefer broad SDM or narrow SDM (by which I really mean the *continuation* of narrow SDM).^10^ Importantly, I am not saying that the clinician should ask this question (or subsequent questions) using the jargon of “broad SDM” and “narrow SDM.” Instead, it would probably be clearer to begin by saying something like “different people prefer making decisions in different ways; do you want me to participate in your decision-making in ways beyond providing information?” However, this paper will continue using the jargon for the sake of brevity.

Given my proposal’s aforementioned timeline, choosing narrow SDM entails choosing that the clinician merely continues providing basic clinical information (e.g., on how the patient’s medical condition evolves after their choice of narrow SDM). It seems likely that many patients would choose narrow SDM. For example, imagine a patient who has already made up their mind that they want palliative care, so they do not want to revisit this decision, and imagine a patient who sees broad SDM as suitable for them and their spouse but not for them and their clinician.

On my approach, the broad SDM that is offered is bound by a central rule (which should be explained to the patient): a clinician who is engaging in broad SDM must have in mind the overarching intention of helping the patient choose a treatment option as rationally as possible (i.e., maximizing alignment with their values), except at times when the clinician is challenging the patient’s values (which I will discuss later). The clinician must not do things like intentionally steer the patient towards a treatment option that the clinician selected because it best aligns with the clinician’s values.^11^ Note that this paper defines “values” loosely. For example, values can be core desires, goals, hopes, purposes, or principles. As a brief aside, I take it as a given that SDM must not involve deception, coercion, or aggression.

Like with narrow SDM, it seems likely that many patients would choose broad SDM. As Anthony Back et al. explain about their study on patients seeking assisted dying, “When the patients in this study approached their clinicians about [assisted dying], they were usually looking for more than just a prescription. They were looking for someone with whom they could build a therapeutic alliance – a person who could act as a sounding board or guide them through the dying process” (Back et al. 2002, 1263). More generally, studies show that most patients prefer broad SDM over narrow SDM (Kon 2010).

Next, my approach dictates that if the patient chooses broad SDM over narrow SDM, the clinician should ask some follow-up questions to determine exactly what type of broad SDM the patient wants. Consider the following four follow-ups. First, “who do you want to guide our discussion?” The patient can answer in a binary way, or they can choose for this to vary by topic (Charles et al. 1999). Second, “would you be OK with me pushing back on some of your views about whether a treatment option aligns with your values?” If the clinician does so, this should be done either because they suspect that the views are false or because they want to make the patient critically reflect on the views. Third, “would you be OK with me pushing back on some of your values?”^12^ If the clinician does so, this should be motivated by the concern that the values are inconsistent, non-autonomous, inauthentic, unexamined, etc. (rather than the concern that the values differ from the clinician’s).^13^ Here I have in mind cases like a disabled patient leaning towards assisted dying because of potentially inauthentic internalized ableism.^14^ Fourth, “do you want me to provide a recommendation if I have one?” Importantly, many of the patients in question would probably say yes (Baylis and Downie 2001; Gómez-Vírseda et al. 2020).

The last question warrants five points of elaboration from me. First, if the clinician provides a recommendation, they should also provide their reasons for it (Baylis and Downie 2001). This should include whose values it is based on (Jacobsen et al. 2018), and this should be the patient’s values (unless they request otherwise).^15^ Second, the clinician should not frame their recommendation as an imperative, command, order, etc. Third, the clinician should explain that the patient can reject the recommended treatment option without facing retaliation, and the clinician will support them in whatever they choose (even if it contradicts the recommendation) (Entwistle et al. 2010; Hempeler et al. 2024). Fourth, the clinician should refrain from providing a recommendation if they feel insufficiently confident regarding which treatment option best aligns with the patient’s values. If the patient nevertheless requests a recommendation, hopefully the clinician can become sufficiently confident via further dialogue. If the patient wants a recommendation but little to no dialogue,^16^ my proposal encourages unconfident clinicians to refuse to provide one. Fifth, regardless of whether the patient consents to receiving a recommendation, if the clinician says something that could be interpreted as a recommendation, they should clarify whether it is one. This is necessary for decreasing misunderstandings; studies show large gaps between whether a clinician believes that they provided a recommendation and whether a patient believes that they received one (Xafis et al. 2015).

Let us take a step back. Although the above follow-up questions matter, the rest of this paper will focus on the narrow vs. broad distinction for the sake of simplicity (but most of the analysis will cross-apply to the follow-ups). On my proposal, there are four rules regarding how a clinician should offer the choice between narrow SDM and broad SDM.^17^ First, the clinician should present these SDM options as neutrally and non-directively as they can (which is crucial for avoiding unduly influencing the patient’s SDM choice). For example, neither narrow SDM nor broad SDM should be framed as the default option, and the clinician should not recommend an SDM option. Second, the clinician should explain that there are no punishments or rewards for choosing a specific SDM option. In particular, no one would punitively worsen the patient’s healthcare. Third, the clinician should explain that the patient can change their mind about the SDM options at any point during SDM.^18^ They can go from narrow SDM to broad SDM, vice versa, or from one type of broad SDM to another type (e.g., by changing their answer to the recommendation question).^19^ Because many patients are unassertive and easily intimidated, the clinician should actively encourage the patient to speak up if they want things done differently (Guadagnoli and Ward 1998; Charles et al. 1999; Joseph-Williams et al. 2014). Fourth, the clinician should provide the patient with as much information about the SDM options as is necessary for informed consent. Now, what information is this?

There are at least five types. The first is the basics of the SDM options (e.g., the techniques involved in broad SDM). This could be illustrated via examples. The second is any SDM-related information that the patient requests. The third is the fact that the clinician is not an expert on the question of which treatment option best aligns with the patient’s values. For example, the clinician can say “I am the expert on the medical facts, but you are the expert on you” (Ubel et al. 2017, p. 37). The fourth, if applicable, is the clinician’s conflicts of interest or relevant biases (e.g., personal opposition to assisted dying), which could seep into how they engage in SDM (Quill and Brody 1996).^20^ The fifth is the main pros and cons of the SDM options. Below, I speculate about what these may be.

The main pros of narrow SDM seem to be that it may make some patients feel more in control of their decision-making (Smith and Pettegrew 1986) and that it may decrease the probability of the clinician influencing the patient in potentially undesirable ways (which I will explore later) (Cribb and Entwistle 2011). The main cons seem to be two-fold.^21^ First, narrow SDM may make the patient’s decision-making less rational (compared to broad SDM).^22^ As Sarah Raskoff summarizes, “recent findings in behavioral economics call into question the extent to which people, left to their own devices, succeed in autonomously or rationally choosing for themselves” (Raskoff 2022, p. 948). In particular, people are subject to a wide range of cognitive biases, including framing effects, loss aversion, and status quo bias (Raskoff 2022). For a particularly pertinent example, consider how “a more-is-better heuristic might influence seriously ill patients who say ‘yes’ when asked if they ‘want everything done,’ even when ‘everything’ might be undesirable” (Epstein and Peters 2009, 196).

The second main con seems to be that narrow SDM can cause suffering (e.g., anxiety) for some patients (Elwyn et al. 1999). On their own, some patients struggle to choose a treatment option because they are unconfident, conflicted, or anticipating self-blame if things go wrong (Entwistle et al. 2010). In short, narrow SDM puts a bigger burden on patients (Gómez-Vírseda et al. 2020). This can be particularly problematic in EOL cases because of the many preexisting burdens (Pujol et al. 2025).

Next, there seem to be two main pros of broad SDM. First, the clinician can help the patient decide more rationally (Epstein and Gramling 2013; Ubel et al. 2018; Peterson et al. 2021). Specifically, “the imagining, reasoning and communicative skills of clinicians… might be used to overcome the varied impairments and challenges that can affect people’s decision-making” (Entwistle et al. 2012, p. 420).^23^ For example, the clinician can tell the patient that part of their decision-making may be distorted by a cognitive bias (Swindell et al. 2010).^24^ Spotting such distortions may be easier for a clinician who has engaged in broad SDM many times (unlike the average patient). However, the clinician should acknowledge that they also have cognitive biases. Again, it may be clearer for the clinician to explain these things without using jargon such as “cognitive bias.” Also, separate from the terminology question, note that a clinician can flag an apparent flaw in a patient’s decision-making without expressly attributing this flaw to a particular cognitive bias.

Furthermore, broad SDM often involves back-and-forth dialogue, and this matters because studies show that human reasoning works best in such contexts (Mercier and Sperber 2011). Moreover, broad SDM gives the clinician more opportunities to discover and correct the patient’s misunderstandings (especially subtle ones that survived the initial information disclosure). Lastly, broad SDM gives the clinician more opportunities to help the patient regulate their emotions during their decision-making. For example, broad SDM involves “sharing the cognitive load,” which can decrease anxiety associated with complex cognitive tasks (Epstein and Gramling 2013, 103 S). This matters for rationality because strong emotions can worsen decision-making and communication (Ubel et al. 2017; Feder and Firn 2019). This is especially important in EOL cases because these cases often involve emotional turbulence such as grief and anguish (Pujol et al. 2025).

The second main pro seems to be that broad SDM allows the clinician to directly help the patient clarify their values. The relevant problem is that “the patient’s values… are often inchoate, and the patient may only partially understand them” (Emanuel and Emanuel 1992, 2222). One of the main reasons for this is that the patient’s medical situation can involve new experiences that disrupt their value system (Cribb and Entwistle 2011). The relevant solution is that the clinician can help the patient “construct, check and prioritize” their values (Cribb and Entwistle 2011, p. 212).^25^ This can involve helping them rebuild their sense of self (Foo et al. 2024).

The main cons of broad SDM all relate to how the clinician may influence the patient. Consider the following three concerns. First, the patient may arationally defer to the clinician because of authority bias. Some patients habitually follow the guidance of trusted healthcare professionals (Entwistle et al. 2008).^26^ Furthermore, the dynamics of broad SDM are often shaped by the patient’s perception of the clinician as an officially legitimized expert (Charles et al. 1999), and “These clinical encounters also occur in a hierarchical setting – the physician with her medical degree and white coat, the patient with, perhaps, a high school education and street clothes” (Ubel et al. 2017, p. 36).^27^

Second, intentionally or unintentionally, the clinician may arationally influence the patient by triggering availability bias, salience bias, framing effects, priming effects, etc. For example, “Physicians subtly influence patients’ preferences by framing outcomes in positive (survival) vs negative (mortality) ways or by presenting a favored option first, making it seem like the middle of the road, highlighting its benefits, and even using a different tone of voice” (Epstein and Peters 2009, 196). Such techniques can operate outside of the patient’s conscious awareness.

Third, the clinician may influence the patient in ways that are driven by unconscious paternalism (Emanuel and Emanuel 1992; Cribb and Entwistle 2011; Engelhardt et al. 2016; Childress and Childress 2020). For example, the clinician may unknowingly steer the patient towards the treatment option that best aligns with the clinician’s views on the good life (Charles et al. 1999). The clinician can try to avoid doing so, but many methods of steering operate outside of one’s conscious control (Smith and Pettegrew 1986).

To be clear, none of the above three cons are *unique* to broad SDM. In narrow SDM, a patient can (i) arationally defer to a clinician (despite the clinician being merely informative), (ii) have their cognitive biases triggered by the clinician (e.g., via the order in which information is presented), and (iii) be influenced in ways that are driven by unconscious paternalism. After all, even if the clinician tries their best to seem neutral, the way they present information can *suggest* (accurately or inaccurately) that they favor a particular treatment option (Geurts et al. 2022). However, these cons do seem less likely in narrow SDM (that accords with my proposal).

To conclude this section, I acknowledge that time constraints may make it impractical for a clinician to inform a patient about the above pros and cons in as much detail as I have provided. I leave open how much detail could be dropped without precluding informed consent.^28^ Presumably, the specifics would depend on the patient (in particular, on what information is material to them). Ultimately, even if the above disquisition of my proposal ends up being an unrealistic ideal, it still matters because ideal theory can advance non-ideal theory (Erman and Möller 2022). Now, before directly arguing that following my proposal would not wrong patients, it is worth providing a brief rationale for it.

## The rationale for my proposal

There are two main reasons for allowing patients to choose from the SDM options. The first is that allowing this aligns with the principle of respect for autonomy, which is “a norm of respecting and supporting autonomous decisions” (Beauchamp and Childress 2013, 13). Both narrow SDM and broad SDM have major pros and major cons. In the relevant cases, neither is clearly better than the other. So, respecting a patient’s autonomy means letting *them* weigh the pros against the cons and choose accordingly.^29^ For illustration, imagine a patient weighing an increased likelihood of choosing the treatment option that best aligns with their related values against an increased likelihood of being influenced in ways that they see as undermining their autonomy (which would go against other values of theirs). It is commonly accepted that the principle of respect for autonomy obliges clinicians to give capable patients a wide range of latitude when it comes to choosing how to make their healthcare decisions. For example, capable patients are allowed to choose between two treatment options by flipping a coin (even though this decision procedure is rationally suboptimal). Of course, the fact that the principle of respect for autonomy supports allowing patients to choose from the SDM options does not settle the broader question of whether this choice should be given, all things considered, but this fact is very significant for this broader question because the dominant view in bioethics is that this principle is of paramount importance (Campbell 2017).

The second reason is more pragmatic. As Alan Cribb and Vikki Entwistle explain, “It is generally likely to be more courteous and more effective for a health professional to go with the grain of what the patient wants in terms of communication and engagement” (Cribb and Entwistle 2011, p. 214). Relatedly, going with the grain can improve the relationship.^30^ The available evidence suggests that fulfilling a patient’s communication preferences tends to increase their trust in, satisfaction with, and endorsement of the clinician in question (Krupat et al. 2000, 2001; Swenson et al. 2004). Among other reasons, this matters because having a close patient-clinician relationship can advance a patient’s autonomy and health outcomes (Entwistle et al. 2010; Kelley et al. 2014; Wampold 2021).

## The likeliest objection to my proposal

Many theorists would object to my proposal by arguing that, for the EOL treatment decisions in question, clinicians should offer only narrow SDM because broad SDM would often (if not always) unduly influence patients. Generally speaking, the narrow-only vision of SDM seeks to protect patients from undue influence (Childress and Childress 2020). Among other reasons, undue influence matters because consent that is motivated by it is invalid (Ploug et al. 2012). So, providing euthanasia to someone who chose it because of undue influence constitutes killing someone without their valid consent, which seems ethically egregious because valid consent is what morally transforms a killing from impermissible to permissible. Now, how can broad SDM unduly influence patients? Based on the academic literature, I foresee critics raising two main ways (which are not mutually exclusive).^31^

First, some critics would argue that broad SDM unduly influences patients when it involves “nudges,” which can be loosely defined as “deliberate changes to and designs of people’s choice environments – the ways in which options are presented or framed – in attempts to predictably steer those people in specific directions” (Schmidt and Engelen 2020, 2). The particularly worrisome nudges (which I will focus on from here on) are those that “bypass the target’s capacity for rational deliberation” (Noggle 2025).^32^ In light of my earlier discussion of how clinicians can arationally influence patients, I concede that broad SDM can involve such nudges. This matters because many theorists argue that it is immoral for clinicians to nudge patients in this way (Engelen 2019), especially for EOL treatment decisions (Saghai 2013). To be charitable to the narrow-only side of the debate, I grant for the sake of argument that narrow SDM (that accords with my proposal) would not involve immoral rationality-bypassing nudges. Also, I leave open the thorny question of exactly what kind of intention is necessary for an influence to count as a nudge (Hansen 2016).

Second, some critics would argue that broad SDM unduly influences patients when it involves recommendations. The narrow-only vision of SDM “requires that physicians withhold their recommendations because they might bias the patient. The physician should objectively answer questions but should avoid influencing the patient to take one path or another, even if the physician has strong opinions or if the patient asks for advice” (Quill and Brody 1996, 764). However, this general concern does not explain why the biasing would be undue, and the concern clearly does not justify withholding recommendations for *all* treatment decisions. After all, a clinician can permissibly recommend a cast for a broken arm. So, critics of my proposal need a specific justification for withholding recommendations for the EOL treatment decisions that are within the purview of my proposal.

Enter Yan Zhou and Wayne Shelton. They make the following argument:

Discussions regarding end of life, particularly those that may involve [assisted dying], are unlike discussions about treatment options for other diagnoses where recommendations can be given about what the best or the most comfortable choice would be. Each person has his/her own sense of what the end entails and what provides comfort and these end of life decisions should be made solely by the individual based on these considerations. For some, it may be a sense of control of when and where they will die which [assisted dying] provides; for others, it may simply be living whatever length of time they have left in the most painless manner. While physicians have an obligation to inform, the ultimate decision maker is the patient (Zhou and Shelton 2020, 237).

In short, “Since decisions about death and dying are extremely personal, physicians should not participate in recommending some options over others” (Zhou and Shelton 2020, 235). Furthermore, Zhou and Shelton’s argument can be buttressed by an argument about epistemic access:

the desires, aspirations, needs, and values of the [patient]… are strictly subjective – they belong to the [patient] and only the [patient] has direct access to them. A [clinician] does not have the knowledge of a [patient’s] inner world. For this reason, it is difficult for [the clinician] to place themselves in the [patient’s] position and tell what would be the best for them. Therefore, [some theorists] believe that the best thing a [clinician] can do to the [patient] is to refrain from providing any advice or recommendations [for highly value-sensitive treatment decisions] (Chańska 2022, p. 385).

I altered this quote to generalize away from its immediate context: genetic counseling. Now, I have two responses to this objection.

## Response #1

Both halves of the objection fail to prove that the influences in question are undue. I will begin with the half about nudges. The common criticisms of clinician nudges do not apply to nudges that accord with my proposal. Recall that my proposal requires the clinician to engage in broad SDM with the overarching intention of helping the patient choose a treatment option as rationally as possible. Nudges that are driven by this intention do not “impose a specific conception of the good on nudgees,”^33^ “intentionally [treat] others as puppets on strings” (Engelen 2019, 50–51),^34^ or “substitute the nudger’s judgment of what should be done for the nudgee’s own judgment” (Hausman and Welch 2010, 130). Also, given the aforementioned disclosures, the patient would be aware of the types of ways in which they are being nudged, which addresses the concern that “nudging often occurs without the nudged being aware they are being nudged” (Dworkin 2020).^35^ Again though, the clinician should avoid the controversial jargon of “nudge.”

Another common criticism of clinician nudges is that they violate the reflective reasoning component of patient autonomy by inducing bad reasoning (Engelen 2019).^36^ However, some nudges advance this component of autonomy by inducing good reasoning (Engelen 2019),^37^ and this is true for nudges that accord with my proposal.^38^ For example, “if physicians have good reason to believe that patients are falling prey to the availability heuristic… and thus making decisions that do not promote their goals, then the physician might counteract this tendency by making other examples more vivid” (Swindell et al. 2010, p. 262). At the very least, such nudges do not *worsen* reasoning.^39^

One might counter by arguing that a clinician who has the overarching intention that my proposal requires could intentionally nudge a patient towards a treatment option – which the clinician believes best aligns with the patient’s values – in a way that induces *procedurally* bad reasoning. However, on my proposal, this would happen very rarely (if not never). In cases where the patient is on the fence regarding the treatment decision (and thus substantially susceptible to the nudges in question), the clinician probably would not immediately know which treatment option best aligns with the patient’s values (for aforementioned reasons).^40^ So, the best way for the clinician to help the patient choose rationally would be to steer them (via nudges and/or otherwise) towards rational thought *processes* (e.g., carefully considering all the treatment options) rather than towards the maximally rational treatment option (which the clinician would not know).

If engaging in broad SDM makes the clinician learn which treatment option is maximally rational for the patient, my proposal directs the clinician to simply provide a recommendation and their reasons (rather than nudging the patient towards this option). Of course, if the patient told the clinician to refrain from recommendations, the clinician should comply, and they should not nudge the patient towards the option because it would be reasonable to infer that the patient would not want them to do so.^41^ Furthermore, even if a clinician *did* nudge a patient towards a maximally value-aligned treatment option by inducing procedurally bad reasoning, it is unclear whether this would violate the patient’s autonomy (understood holistically); “When heuristics-triggering nudges violate [the reflective reasoning] component of people’s autonomy, they can actually increase the extent to which people act according to their more fundamental values and goals and thus foster self-rule, another component of autonomy” (Engelen 2019, p. 55).^42^

Next, I have five replies to Zhou and Shelton’s recommendation argument. First, Zhou and Shelton provide no reasons for why recommendations for extremely personal decisions are impermissible, and there are many counterexamples. Imagine a good friend recommending proposing to one’s partner, or a loving spouse recommending another round of chemotherapy. As Entwistle et al. explain, “narrow understandings of shared decision-making… can inappropriately idealize ‘uninfluenced’ patient choice and, by doing so, undermine common-sense recognition that preferences developed independently are not necessarily better than treatment preferences developed in collaboration” (Entwistle et al. 2012, p. 419).

Second, providing a recommendation does not change the fact that “the ultimate decision maker is the patient” (Zhou and Shelton 2020, 237). The patient can ignore the recommendation if they wish.^43^ In fact, *truly* treating the patient as the ultimate decision-maker requires giving them more control over the decision-making process (as my proposal does).^44^

Third, unless the patient requests otherwise, the clinician would provide their recommendation based on considerations like the patient’s “sense of what the end entails” (Zhou and Shelton 2020, 237). If the patient makes their treatment decision based on such a recommendation, they would still be deciding based on the considerations that Zhou and Shelton endorse. Furthermore, Zhou and Shelton provide no reasons for why it would be bad for a patient to decide based on other considerations.

Fourth, the recommendation argument is right that clinicians lack direct access to patients’ values, but there are many reasons for why this is not a dealbreaker for my proposal. To begin with, a clinician who follows my proposal would know a lot about the patient’s values despite lacking direct access. Recall that I am defending my proposal only in cases where the clinician already has a knowledgeable relationship with the patient, and engaging in broad SDM (that accords with my proposal) would involve the clinician learning even more about the patient’s values (e.g., by beginning broad SDM with a question like “which of your values do you see as relevant to this treatment decision?”). Of course, patients’ values can change over time, so clinicians should be on the lookout for this during broad SDM. Next, note that some patients *also* lack direct access to their values. As previously discussed, a patient’s values can be unclear to them, in which case they may not be epistemically superior to the clinician when it comes to their values. Lastly, when the patient’s values *are* clear to them, providing a recommendation (that accords with my proposal) would involve the clinician making the most of the patient’s direct access by reaching the recommendation in collaboration with them. Ideally, both parties would bring unique insights to the table, and broad SDM would synthetize these insights. On the clinician side, they could bring extensive experience helping patients apply their values to EOL treatment decisions, so the clinician could excel at this application in ways that the patient would not.

My fifth and final reply is that Zhou and Shelton discuss only the supposed *upsides* of a clinician refraining from providing EOL recommendations, so they ignore the *downsides* of a clinician refusing to provide a recommendation that a patient requests (when the clinician has one). In particular, this refusal would jeopardize informed consent. As Francoise Baylis and Jocelyn Downie argue,

providing patients… with a professional recommendation is an important part of the informed choice process, especially when the patient specifically asks for such information, thereby clearly indicating that she considers this information material in evaluating her treatment options. The physician has an obligation to disclose… information that the patient indicates is material (Baylis and Downie 2001, p. 21).

Furthermore, refusing to provide a desired recommendation can significantly emotionally harm a patient (Beach and Sugarman 2019). For example, they can feel neglected or even abandoned (Entwistle et al. 2010; Childress and Childress 2020).

## Response #2

The more fundamental (and philosophically interesting) reason for why the nudges and recommendations that my proposal allows would not be undue influence is that the patient’s informed and voluntary selection of the relevant SDM option would constitute fully morally transformative consent (i.e., consent that makes an undue influence not undue). For example, I am happy to concede that if a patient does not consent to receiving a recommendation for one of the EOL treatment decisions in question, then providing one (especially for or against assisted dying) can be undue influence (although not for Zhou and Shelton’s reasons).^45^ However, providing one would not be undue if the patient validly consents. By loose analogy, someone’s valid consent to hypnosis makes it no longer undue to perform it on them, despite the rationality-bypassing (Wilkinson 2013).

The story is more complex for nudges. On my proposal, the patient does not directly consent to receiving nudges; they consent to broad SDM (and then to a type thereof) after the clinician informs them how the associated nudge techniques work (albeit without using the word “nudge”). Again, I am happy to concede that in the absence of this consent, nudging a patient vis-à-vis one of the EOL treatment decisions in question can be undue influence. However, valid consent to a type of broad SDM morally transforms the discrete techniques that comprise the practice. By analogy, unconsensual surgery is assault (ethically speaking), and valid consent to a surgery makes it not undue to perform the discrete techniques that comprise the practice (e.g., the specific incisions), even if the patient did not consent to each technique individually (but was informed of the types of techniques involved). Similarly, someone can validly consent to the “open-endedness of [their] neighbour ‘helping themselves’ to [their] lawn mower” despite not knowing “all the possible ways in which their neighbour might use their lawn mower” (Vassilicos and McGann 2025, 1212).^46^ Returning to nudges, Timothy Wilkinson argues that consent makes manipulative nudges consistent with autonomy (Wilkinson 2013), and I would add that the same is true for undue nudges that are indirectly consented to (via direct consent to an overarching practice).

Ultimately, given the sweeping morally transformative power of consent,^47^ I do not have to perform the difficult task of determining the full list of broad SDM techniques that would be undue influence – for one of the relevant EOL treatment decisions – in the absence of consent, nor do I have to determine *why* they would be undue.^48^

One might counter my second response by arguing that there are three main ways in which a patient’s consent to broad SDM (or a type thereof) could fail to be fully morally transformative (in which case the consent would not make the undue influences not undue).^49^ First, the consent could be driven by authority bias. Perhaps the arational tendency to defer to the guidance of authority figures can manifest as an arational choice to receive such guidance. Second, the patient could lack information that is material to the SDM options. For example, some patients pursue guidance partly because they do not recognize the pertinence of their preferences (Ubel et al. 2017). Relatedly, “Many patients believe ‘doctor knows best’, and that their own knowledge is superfluous to the decision-making process, being trumped by medical expertise and experience” (Joseph-Williams et al. 2014, p. 303). Third, the consent could be driven by the power imbalance between the powerful clinician and the vulnerable and dependent patient (Guadagnoli and Ward 1998).^50^ In the context of SDM, Natalie Joseph-Williams et al. highlight that many patients want to be seen as a good patient – rather than a difficult patient – to acquire perceived benefits (e.g., getting the clinician “on your side”) and avoid retribution (e.g., worse care) (Joseph-Williams et al. 2014, p. 303). This can make patients passive in decision-making (Childress and Childress 2020). Theoretically, a patient could choose broad SDM because they believe that doing so will make their clinician give them better care.

However, none of these possibilities meaningfully threaten my proposal. Regarding authority bias, I have three replies. First, the odds of a patient choosing broad SDM (or a type thereof) because of authority bias are heavily mitigated by my proposal’s required disclosures (e.g., regarding authority bias itself and the patient’s superior expertise on their values). Second, these odds are also heavily mitigated by the clinician’s explicit neutrality about the SDM options. If my proposal allowed clinicians to recommend broad SDM, then yes, many patients might choose it because of authority bias, but my proposal disallows this. Third, informed and voluntary consent can be fully morally transformative despite being driven by authority bias. For examine, imagine a normal case of a clinician recommending a surgery. If the patient consents to the surgery largely because of authority bias, it seems that this would not make it at all undue for a surgeon to perform the surgery.^51^

As a brief aside, it is worth noting that analogs of this third reply are also true for cognitive biases other than authority bias. For example, imagine a clinician bringing up a surgery before the other treatment options (perhaps because of unconscious paternalism). If the patient consents to the surgery largely because of the primacy effect, it seems that this would not make it at all undue for a surgeon to perform the surgery. Analogously, if a patient consents to broad SDM largely because a clinician brought it up before narrow SDM (perhaps because of unconscious paternalism), this would not prevent the consent from being fully morally transformative. Things might be different if a clinician *intentionally* triggered a cognitive bias to steer a patient towards broad SDM (paternalistically or otherwise), but my proposal forbids this.

Regarding insufficient information, I have two replies. First, my proposal requires giving the patient a lot of information about the SDM options (including information that clarifies the pertinence of the patient’s values, preferences, and knowledge).^52^ Second, if my proposal currently overlooks any information that is necessary for informed consent, add it to the disclosures.

Regarding power imbalance, I have a reply with three parts. First, the mere *existence* of a power imbalance between two people does not change whether consent from one to the other is fully morally transformative.^53^ Generalizing a point by David Boonin (in a way he would agree with), I contend that “it’s the pressure and plausible fear of retaliation that generates [the relevant] doubts we might… have about the validity of [a patient’s] consent to [an interaction] with [a healthcare professional]… and not the mere fact of the positional inequality… within their professional relationship” (Boonin 2022, p. 384). By analogy, coercion does not change the voluntariness of consent unless it sufficiently motivates the consent (Olsaretti 2004; Hyman 2013).

Second, if a clinician follows my proposal, it would be extremely unlikely for a patient to consent to broad SDM (or a type thereof) because of felt pressure or fear of retaliation. Because I am defending my proposal only in cases with a trusting relationship, the patient would probably believe the clinician when they say that there is no risk of retaliation. Furthermore, even if the clinician’s explicit neutrality fails to prevent the patient from inferring that the clinician has a strong desire for them to choose broad SDM, they would have no reason to infer that the clinician would retaliate against them for choosing otherwise. Admittedly, it seems *possible* that the patient would choose broad SDM in a misguided attempt to avoid offending the clinician, but consent that is motivated by a desire to avoid offence seems fully morally transformative when the other party credibly denies that they would be offended and when the consenter does not fear the offence causing retaliation.

Third, even if the patient were to choose an SDM option based on inferring what would be best for the clinician (regardless of whether the patient fears retaliation), they would probably choose *narrow* SDM because it takes less time for the clinician. Some patients’ SDM-related choices are influenced by pity for busy-seeming clinicians and guilt about taking up their time (Joseph-Williams et al. 2014). If such considerations make a patient choose narrow SDM, this may be in some sense unfortunate,^54^ but it is not particularly important whether consent to narrow SDM is fully morally transformative, given that narrow SDM (that accords with my proposal) does not seem to involve any undue influence in need of being morally transformed by consent. Presumably, the narrow-only side of the debate would agree with this.

## Conclusion

As shown, there are cases in which a patient would not be wronged by a clinician letting them choose from a range of SDM options for an EOL treatment decision. Giving a patient this SDM choice advances their autonomy by giving them more control over how they make this treatment decision, and it may improve the patient-clinician relationship. It is understandable that critics would fear undue influence in the form of nudges and recommendations, but following my proposal would preclude the most problematic types of these influences and ensure that the patient’s SDM choice constitutes fully morally transformative consent that permits whichever influences would be undue in the absence of this consent.

At this point, one might be wondering why I have argued for an absence of wronging rather than arguing for all-things-considered permissibility or even obligatoriness. This is because justifying permissibility or obligatoriness would require exploring the impacts on the clinician and third parties. For example, discussing assisted dying with a patient can make a clinician feel emotionally drained, isolated, and afraid of judgement from others (Zhou and Shelton 2020).^55^ Also, some clinicians may conscientiously object to engaging in broad SDM that accords with my proposal. Lastly, there is an opportunity cost. For broad SDM, “Because the information exchange is two way rather than one way, as are processes of deliberation and decision-making, this approach is likely to take more time,” and “Time spent by physicians with a given patient… depletes the time available to them for other needy patients in their practice” (Charles et al. 1999, p. 655). This opportunity cost can also harm the clinicians themselves because they are often unreimbursed for engaging in SDM (Joseph-Williams et al. 2014). Ultimately then, I am not claiming that clinicians *should* follow my proposal; I am just denying that doing so would unduly influence patients. However, I contend that I have shown that my proposal is promising enough to warrant empirical testing (e.g., to confirm the more speculative parts of my analysis and determine the severity of my proposal’s downsides).

## Footnotes

1 For the sake of simplicity, I will not discuss SDM that involves additional people (e.g., the patient’s family members or other members of their care team).

2 I agree with theorists who prefer the phrase “assisted decision making” (Childress and Childress 2020, p. 426), but I am using the phrase “shared decision-making” to align with the most relevant academic literature.

3 There are cases where my proposal supports persuading a patient to request assisted dying, so Canadian law opposes my proposal in these cases.

4 The United Nations Convention on the Rights of Persons with Disabilities takes a similar approach to supporting the decision-making of some intellectually disabled people (Series 2015).

5 I leave open the question of how often a clinician can know whether these features apply to a case that they are in.

6 These skills include “the moral sensitivity, relational skills, and interpretive capacities necessary to engage with patients in an attuned and supportive manner” as well as “openness to the perspective of others, willingness to revise one’s beliefs, and the ability to co-create meaning in uncertain or conflictual situations” (Pujol et al. 2025, p. 3, 10). Unfortunately, many clinicians lack such skills, especially in EOL contexts (Granek et al. 2013; Visser et al. 2014).

7 If the reader prefers thinking of decision-making capacity as spectral rather than binary, please attribute *sufficient* decision-making capacity to the patients who are within the scope of this paper.

8 EOL patients can lack decision-making capacity for multiple reasons, including neurocognitive disorders (e.g., dementia) and emotional instability (Pujol et al. 2025).

9 Many healthcare organizations (e.g., medical schools) have been doing so (Dewi et al. 2024; Moezzi et al. 2024).

10 The clinician should ask this directly (rather than inferring how the patient would answer) because studies have long shown that clinicians are “poor judges of the decision-making preferences of their patients” (Deber 1994, p. 424). To be fair, I acknowledge that many patients have unspoken decision-making contracts with their clinicians (Joseph-Williams et al. 2014), but it would be much less ethically risky if these contracts were spoken. Next, one might be wondering about the appropriateness of a clinician providing information about the treatment options and then saying nothing about SDM, thereby putting the burden on the patient to raise the topic. This is prima facie problematic because it deprives many patients of a meaningful SDM choice. Many patients would be unaware that broad SDM is an available option, would assume that it is the clinician’s job to raise it, or would be too uncomfortable raising it.

11 Clinicians should actively reflect on whether they are steering patients in this way (Pujol et al. 2025).

12 This would not have to be done from the perspective of the clinician’s value system. After all, you can challenge someone’s values despite agreeing with them.

13 It is beyond the scope of this paper to explore the debate over whether a value can be non-autonomous merely by virtue of its content (rather than properties like its causal history).

14 To be clear, I am not saying that a clinician can reliably independently determine whether one of a patient’s values comes from internalized ableism. However, I contend that a clinician can suspect that internalized ableism is playing a role and then either explore this possibility with the patient or push back on the value in question without mentioning internalized ableism.

15 The patient could ask the clinician to provide a recommendation based on the clinician’s values. If the patient asks “what would you do?”, the clinician should ask whether they are seeking an answer based on the clinician’s values or an answer based on the patient’s values (Baylis and Downie 2001, 22–23).

16 Among other reasons, a patient can want this because they prefer passivity or believe that they are not entitled to participate in their healthcare decisions (Joseph-Williams et al. 2014).

17 I am open to this choice being offered for a well-defined *set* of EOL treatment decisions that are all interconnected, high-stakes, highly value-sensitive, and lacking clinically superior options.

18 The clinician should remind the patient of this fact if broad SDM extends across multiple sessions or if the patient seems uncomfortable with something the clinician is doing.

19 A patient’s SDM preferences can vary over time (Dowie 2002).

20 Understandably, some patients may react to this disclosure by choosing narrow SDM.

21 It is beyond the scope of this paper to explore the extent to which these cons can be mitigated by the patient doing something like broad SDM with someone other than the clinician.

22 This is especially true for “People with limited education and/or health literacy, and people with learning disabilities” (Entwistle et al. 2012, p. 419).

23 My claim is not that clinicians are better at these skills than patients are (although this may well be true). Instead, my claim is that combining a clinician’s skills with a patient’s skills has a lot of potential to increase rationality.

24 For illustration, consider the following case: “a dialysis patient who recently had a leg amputation and was also left by her romantic partner may fall prey to the impact bias… and choose to forego dialysis because she thinks that she will never get over this loss. Although it may not be clear what choice is in this patient’s best interests (or what her long-term goals are), the clinician should inform her about how the impact bias works (that people tend to overestimate how long and intense their pain will be when something bad happens to them) and make her aware that it may be influencing her decision” (Swindell et al. 2010, p. 262).

25 As an added bonus, this can strengthen the patient-clinician relationship (Epstein and Gramling 2013; Foo et al. 2024).

26 To be clear, deferring based on trust *can* be rational.

27 Consider the Milgram experiments (Milgram 1963).

28 Informed consent does not necessitate a perfect understanding of the options (Eyal 2025).

29 This reason was inspired by an argument from Dowie (Dowie 2002).

30 This claim aligns with evidence on supported decision-making for intellectually disabled people (Series 2015).

31 Some critics would object that challenging a patient’s values unduly influences them, but Ezekiel and Linda Emanuel have already thoroughly rebutted this objection (Emanuel and Emanuel 1992).

32 See Blumenthal-Barby as well as Ploug and Holm (Blumenthal-Barby 2012; Ploug and Holm 2015).

33 See Tengland and White (Tengland 2012; White 2013).

34 See Hausman and Welch, Grüne-Yanoff, and Schmidt (Hausman and Welch 2010; Grüne-Yanoff 2012; Schmidt 2017).

35 For more on this concern, see Blumenthal-Barby (Blumenthal-Barby 2012).

36 See Ploug and Holm (2015).

37 See Hanna (Hanna 2015).

38 For a full account of how nudges can “combat failures of rationality in end-of-life decision-making, and promote a relational autonomy,” see Megan Wright (Wright 2018, 1067).

39 Consider Jillian Craigie’s “discursive control account of freedom,” according to which “the involvement of others undermines decision-making freedom when it limits discourse about what the preexisting reasons relating to a decision require” (Craigie 2015, p. 398, 403). Nudges that accord with my proposal do not do this.

40 Critics of clinician nudges would fully agree with me on this, given their epistemic concerns (Engelen 2019). For more on these concerns, see White (White 2013).

41 Here and in the previous paragraph, my analysis echoes Blumenthal-Barby’s opposition to clinician nudges in cases where it is unclear which treatment option would be best for the patient, as well as cases where the patient would object to being nudged (Blumenthal-Barby 2021).

42 For more on how clinician nudges can advance patient autonomy, see Blumenthal-Barby (Blumenthal-Barby 2021).

43 If a patient arationally defers to a recommendation after choosing to receive a recommendation despite being informed of the risk of arational deference, it seems that they are still the ultimate decision-maker.

44 It is worth clarifying that my proposal does not allow the patient to use their ultimate decision-making authority to transfer this authority to the clinician (not even partially, e.g., by giving them a legally binding veto). I take no position on the ethics of this; I just want to keep my proposal in line with existing laws regarding consent to high-stakes treatment options.

45 I am open to one of the main reasons being a violation of an implicit procedural norm of the relationship. For an account of the connection between such norms and undue influence, see Mindy Chen-Wishart (Chen-Wishart 2006). Relatedly, see Blumenthal-Barby’s work on the relational dimension of undue influence (Blumenthal-Barby 2012).

46 For some theoretical background, see Robert Goodin (Goodin 2024).

47 The other side of the debate has not shown (or even tried to show) that broad SDM involves techniques that are undue in ways that consent cannot eliminate. Furthermore, this would be very hard to show because my proposal safeguards against things like exploitation, domination, oppression, abuse, unfairness, intentional paternalism, and acting in bad faith.

48 In the absence of exceptional circumstances, it would wrong a patient for a clinician to engage in broad SDM in a way that intentionally, knowingly, recklessly, or negligently goes beyond what the patient consented to. If there is a non-negligible risk of the clinician going beyond the scope of the consent despite exercising due care, they should disclose this as another con of broad SDM. If the patient nevertheless chooses broad SDM, it would not wrong them for the clinician to engage in broad SDM despite the risk. By analogy, it can be permissible for a surgeon to perform a surgery despite a small risk of them accidentally doing something that goes beyond what the patient consented to.

49 By loose analogy, if a student consents to sex with their professor to get a better grade, it remains undue for the professor to have sex with them, but it is clearly *less* undue than if they did not consent at all.

50 This imbalance can be exacerbated by social factors like racial differences (Joseph-Williams et al. 2014).

51 It seems to me that two parts of the explanation for this permissibility are the absence of manipulation and the surgeon’s ignorance of the role played by the cognitive bias.

52 Many studies show that SDM is facilitated by patients understanding this pertinence (Joseph-Williams et al. 2014).

53 The counter to my second response implicitly concedes this.

54 However, it is possible for a patient to autonomously choose an SDM option based on what would be best for the clinician (even based on avoiding saddening them). By loose analogy, someone can autonomously choose to donate to charity based on what would be best for the beneficiaries, and, less loosely, an EOL patient can autonomously choose to prolong their life to avoid saddening their children.

55 Such emotional tolls on clinicians can worsen patient-clinician communication (Weiner and Cole 2004).
